# Impacts of Simulated Weightlessness by Dry Immersion on Optic Nerve Sheath Diameter and Cerebral Autoregulation

**DOI:** 10.3389/fphys.2017.00780

**Published:** 2017-10-12

**Authors:** Marc Kermorgant, Florian Leca, Nathalie Nasr, Marc-Antoine Custaud, Thomas Geeraerts, Marek Czosnyka, Dina N. Arvanitis, Jean-Michel Senard, Anne Pavy-Le Traon

**Affiliations:** ^1^UMR Institut National de la Santé et de la Recherche Médicale 1048, Institut des Maladies Métaboliques et Cardiovasculaires, Toulouse, France; ^2^Department of Anesthesiology and Intensive Care, University Hospital of Toulouse, Toulouse, France; ^3^Department of Neurology and Institute for Neurosciences, University Hospital of Toulouse, Toulouse, France; ^4^BNMI, UMR Institut National de la Santé et de la Recherche Médicale 1083, UMR Centre National de la Recherche Scientifique 6214, Centre de Recherche Clinique, University Hospital of Angers, Angers, France; ^5^Toulouse NeuroImaging Center, UMR 1214, Institut National de la Santé et de la Recherche Médicale, Université Toulouse III-Paul Sabatier, Toulouse, France; ^6^Brain Physics Laboratory, Division of Neurosurgery, Department of Clinical Neurosciences, Cambridge University Hospital, Cambridge, United Kingdom; ^7^Institute of Electronic Systems, Warsaw University of Technology, Warsaw, Poland

**Keywords:** transcranial Doppler, cerebral autoregulation, optic nerve sheath diameter, intracranial pressure, dry immersion

## Abstract

Dry immersion (DI) is used to simulate weightlessness. We investigated in healthy volunteers if DI induces changes in ONSD, as a surrogate marker of intracranial pressure (ICP) and how these changes could affect cerebral autoregulation (CA). Changes in ICP were indirectly measured by changes in optic nerve sheath diameter (ONSD). 12 healthy male volunteers underwent 3 days of DI. ONSD was indirectly assessed by ocular ultrasonography. Cerebral blood flow velocity (CBFV) of the middle cerebral artery was gauged using transcranial Doppler ultrasonography. CA was evaluated by two methods: (1) transfer function analysis was calculated to determine the relationship between mean CBFV and mean arterial blood pressure (ABP) and (2) correlation index Mxa between mean CBFV and mean ABP.ONSD increased significantly during the first day, the third day and the first day of recovery of DI (*P* < 0.001).DI induced a reduction in Mxa index (*P* < 0.001) and an elevation in phase shift in low frequency bandwidth (*P* < 0.05). After DI, Mxa and coherence were strongly correlated with ONSD (*P* < 0.05) but not before DI. These results indicate that 3 days of DI induces significant changes in ONSD most likely reflecting an increase in ICP. CA was improved but also negatively correlated with ONSD suggesting that a persistent elevation ICP favors poor CA recovery after simulated microgravity.

## Introduction

Due to the difficulties to perform in-flight experiments in addition to restricted opportunities of spaceflight, models are used on Earth to simulate the effects of microgravity. Dry immersion (DI) is a model of ground-based simulated microgravity (Pavy-Le Traon et al., 2007) where the subject is immersed and separated from the water with waterproof fabric (Navasiolava et al., 2011). This method allows for a rapid fluid migration toward the upper body. DI also removes afferent signals from the support zones and triggers a drop in the activity of the postural muscle system (Gevlich et al., 1983; Navasiolava et al., 2011; Watenpaugh, 2016). Adaptation of the cardiovascular system to microgravity is complex and its underlying mechanisms are not thoroughly understood. Exposure to real or simulated microgravity induces a redistribution of body fluids toward the upper part of the body (Charles and Lathers, 1991). This cranial redistribution observed in astronauts after exposure to microgravity is likely responsible for the elevated intracranial pressure (ICP) reported after long-duration flights (Nelson et al., 2014). The determination of the optic nerve sheath diameter (ONSD) can be used as an indirect marker of increased ICP (Geeraerts and Dubost, 2009; Dubost et al., 2011) and changes in ONSD have been shown to be a reliable surrogate of changes in ICP. Visual impairment has been reported in some astronauts exposed to long duration spaceflights. Indeed, some changes such an increase in ONSD, posterior globe flattening and optic nerve protrusion suggest a potential intracranial hypertension (Kramer et al., 2012), resulting in the Vision Impairment and Intracranial Pressure syndrome (Nelson et al., 2014). Therefore, studies are currently performed to examine and understand the effect of exposure to microgravity on ICP. Exposure to microgravity may also affect cerebral autoregulation (CA). Impaired CA has been proposed as a contributing factor to orthostatic intolerance reported after real or simulated microgravity. However, in-flight data on CA are controversial. An impaired CA could induce a reduction in the phase frequency between cerebral blood flow (CBF) and blood pressure and may lead to presyncope (Blaber et al., 2011). It has been shown that astronauts which spent long-duration flights on the International Space Station had an impaired CA with a decrease in cerebrovascular CO_2_ reactivity (Zuj et al., 2012). In contrast, a study performed on astronauts in a 1 and 2-week spaceflight showed an improved CA with a decreased low frequency (LF) gain (Iwasaki et al., 2007).

The aim of the study was to assess the changes in ONSD after exposure to simulated microgravity by DI and to determine whether these changes may impact CA.

## Materials and methods

### Subjects

Twelve healthy male volunteers participated in this study. None of the participants were smokers or took any medical treatment or drugs. All participants were informed about clinical assessment and gave their written consent. Participants selected for the experiments did not exhibit acute or chronic pathologies, which could affect the physiological data. The volunteers had normal clinical and paramedical examination and laboratory tests (hematology and blood chemistry). This Clinical Trial was conducted in accordance with the principles laid down by the 18th World Medical Assembly (Helsinki, 1964) and approved by the Ethics Committee (CPP Sud-Ouest Outre-Mer I) and the French Health Authorities. The study was conducted by the Institute for Space Medicine and Physiology (MEDES-IMPS) in Toulouse, France.

### General protocol

**Figure 1:**
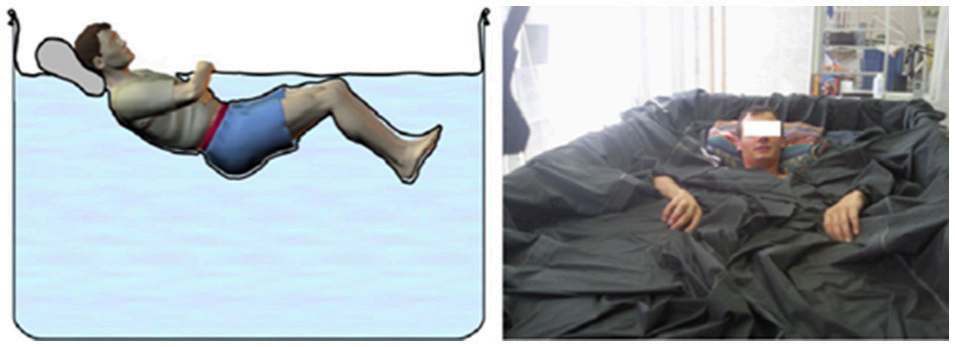
Dry immersion experiment.

The volunteers ensued an ambulatory control period (BDC), 3 days of DI (DI 1–DI 3) and 2 days of recovery (R 0–R+1). Except in DI periods, all subjects remained in ambulatory conditions. During DI, the subject was immersed slowly into the water in supine position (Figure 1). The water temperature was automatically set to 32–34.5°C (thermoneutral) and adjusted if needed for subject's comfort. The study was carried out in a quiet room and the air temperature was approximately 24°C. All subjects remained continually under medical observation. The folds of fabric could be moved easily for the different recordings without affecting the experimental conditions. Once a day and during 15 min, the subject was allowed to get out from the bath for hygiene procedures. The volunteers woke up at 6:30 a.m. and the light was switched off at 11:00 p.m. Each subject had a daily medical follow-up including ABP and heart rate (HR) measurements by permanent staff of MEDES. The flow chart is represented Table 1.

**Table 1:** Study flow chart.

	**Phases**					
	**BDC**	**DI**			**R**	
		**1**	**2**	**3**	**0**	**+1**
Plasma volume	X	X			X	
Ocular ultrasonography	X	X		X		X
Transcranial Doppler	X					X
Blood pressure measurements	X					X

*BDC, baseline data collection; DI, dry immersion; R, recovery*.

### Plasma volume measurements

The plasma volume was estimated by CO-rebreathing method (SpiCO®; Blood tec, Bayreuth, Germany) in the morning (before breakfast) in resting supine position just before the first day of DI (DI 1) and immediately after the end of DI period, just before standing (R 0).

### Ocular ultrasonography

Ocular examination was realized at rest in supine position during BDC, DI 1, DI 3, and R+1 by investigators trained for ocular ultrasonography. A thick layer of gel was applied over the closed upper eyelid. The probe was placed on the gel in the temporal area of the eyelid and adjusted to obtain an appropriate display of the optic nerve into the globe. The prospection was realized in a two-dimensional mode and ONSD was measured 3 mm behind the ocular globe. Right and left optic nerves were assessed and two measures were realized for each eye: a first measure in the transverse plane (horizontal probe) and a second measure in the sagittal plane (vertical probe). The final measure corresponds to the average of the four measures: horizontal right eye, vertical right eye, horizontal left eye and vertical left eye. All the measures of ONSD were validated by an expert (Thomas Geeraerts) blinded from the subject condition. The subjects were separated into 2 groups: “good recovery” and “poor recovery” groups. To define these groups, an arbitrary cut-off value ONSD was established when subjects recovered ONSD values below 20% between BDC and R+1.

### Transcranial doppler ultrasonography and blood pressure measurements

The assessment of CA was realized in supine position and performed during the morning at BDC and R+1. A 2-MHz Doppler probe (EZ-DOP, DWL, Germany) maintained by a headset was placed close to the temporal window in order to obtain signal from the right middle cerebral artery (MCA) and thus to assess relative CBF changes. The MCA was insonated unilaterally at a depth of 50–55 mm. The continuous ABP was non-invasively monitored by a photoplethysmographic monitor (Nexfin–B Meye, the Netherlands).The signals, cerebral blood flow velocity (CBFV) and arterial blood pressure (ABP) were synchronized, acquired with Biopac MP 150 and visualized on the screen of a PC.

### Analysis of cerebral autoregulation by transfer function analysis

Beat-by-beat mean ABP and CBFV were linearly interpolated and resampled at 4 Hz for spectral analysis. Using fast Fourier transform with 50% superposition of segments (Welch algorithm), the mean CBFV and mean ABP time series, beforehand preprocessed, were transformed from the time domain to the frequency domain. A length of 100 s was chosen for data segments and these segments were passed through a Hanning window. The transfer function analysis is a mathematic model of the relationship between changes in input and output signals, respectively beat-to-beat mean ABP and mean CBFV. To predict to what extent ABP has an influence on CBFV, a cross-spectral analysis method was applied. This analysis enabled to obtain three parameters: coherence, gain and phase. The coherence function measured the fraction of output power that was explained by the input power at a given frequency. The coherence was between values 0 and 1. A value close to 1 indicated, a strong linear relationship between the two signals with high signal-to-noise ratio, whereas the coherence approximating with values near zero may suggest a nonlinear relationship, a low signal-to-noise ratio or other variables influencing variables. To validate gain and phase values, it is necessary to obtain coherence value over 0.5 to ensure measures robustness (Claassen et al., 2016). Over a specified frequency range, the gain reflects the relative amplitude between the changes in the two ABP and CBFV signals and the phase is considered as temporal relation between these signals (Zhang et al., 1998). The recordings with phase shift wrap-around were corrected by adding 2 π. The mean values of the transfer function (coherence, phase and gain) were calculated in very low frequency (VLF: 0.02–0.07 Hz), low frequency (LF: 0.07–0.20 Hz) and high frequency (HF: 0.20–0.35 Hz) ranges as previously defined (Zhang et al., 1998, 2009). The mechanisms of CA can be considered to reflect a high-pass filter that dampens slow fluctuations of blood pressure but allows for passing through of rapid oscillations, such as the pulsatile signals of the blood pressure waves. To assess dynamic CA, the previously calculated mean values of the transfer function were assessed in the VLF and LF bands as a measure of the transmission of blood pressure fluctuations on CBFV (Zhang et al., 1998). These signals were processed with the PDL software (Notocord Systems, France).

### Analysis of cerebral autoregulation by the autoregulatory index mxa

CA was also evaluated using the correlation coefficient Mxa, established from the spontaneous variations of mean ABP and mean CBFV. The acquisition of these parameters was described previously. The Mxa index was assessed as follows: firstly, mean ABP and mean CBFV were calculated after specific signal filtering to reduce or remove the influence of noise or artifacts, according to the recent recommendations (Claassen et al., 2016); secondly, 60 consecutive 10-s periods were established to calculate the Pearson's correlation coefficients between mean ABP and mean CBFV and; thirdly, the resulting 60 Mxa correlation coefficient were finally averaged in order to obtain the autoregulatory index Mxa. A Mxa value close to 1 indicated that ABP variations affected changes in CBFV, thus determining a disturbed CA. A Mxa value nearby 0 stated that ABP variations did not impact CBFV variations, suggesting a normal CA (Czosnyka et al., 1996). The threshold of Mxa commonly used to characterize a CA impairment is >0.45 (Brady et al., 2010).

### Statistics

General hemodynamics parameters, transfer function analysis results, ONSD and Mxa data were expressed as mean ± SD. Paired-t test was used for comparisons of data between BDC and R+1. One-way repeated measures analysis of variance was used to compare ONSD values and general hemodynamics parameters and Dunnett method was used for *post-hoc* analysis. Unpaired-t test was performed to compare Mx values in “good recovery” and “poor recovery” groups at BDC-3 and R+1. Correlation Mxa-ONSD and coherence-ONSD were tested by Spearman rank correlation coefficient. All statistical analyses were performed with GraphPrism 7.00. Differences were considered as statistically significant when *P* < 0.05.

## Results

The main characteristics of the 12 healthy male volunteers participating in this study are the following: mean ± SD at BDC, 32 ± 5 years; 177 ± 6 cm; 74 ± 8 kg.

The changes in mean systolic blood pressure (SBP), diastolic blood pressure (DBP), heart rate (HR) were measured at rest in supine position between BDC and R+1. The plasma volume measurements were realized between DI 1 and R 0. We noted no significant changes either in SBP or DBP whereas HR increased after DI (*P* < 0.001). DI induces a significant decrease (17%) in plasma volume (*P* < 0.001) in all subjects (Table 2).

**Table 2:** General hemodynamics parameters.

	**BDC**	**R+1**	***P*-value**
SBP (mmHg)	122.5 ± 13.2	123.8 ± 11.4	0.737
DBP (mmHg)	63.2 ± 7.5	61.9 ± 5.7	0.614
HR (bpm)	59.0 ± 9.2	65.0 ± 10.6	<0.001
	**DI 1**	**R 0**	
Plasma volume (L)	3.73 ± 0.38	3.10 ± 0.30	<0.001

*DBP, diastolic blood pressure; DI, dry immersion; HR, heart rate; SBP, systolic blood pressure*.

**Figure 2:**
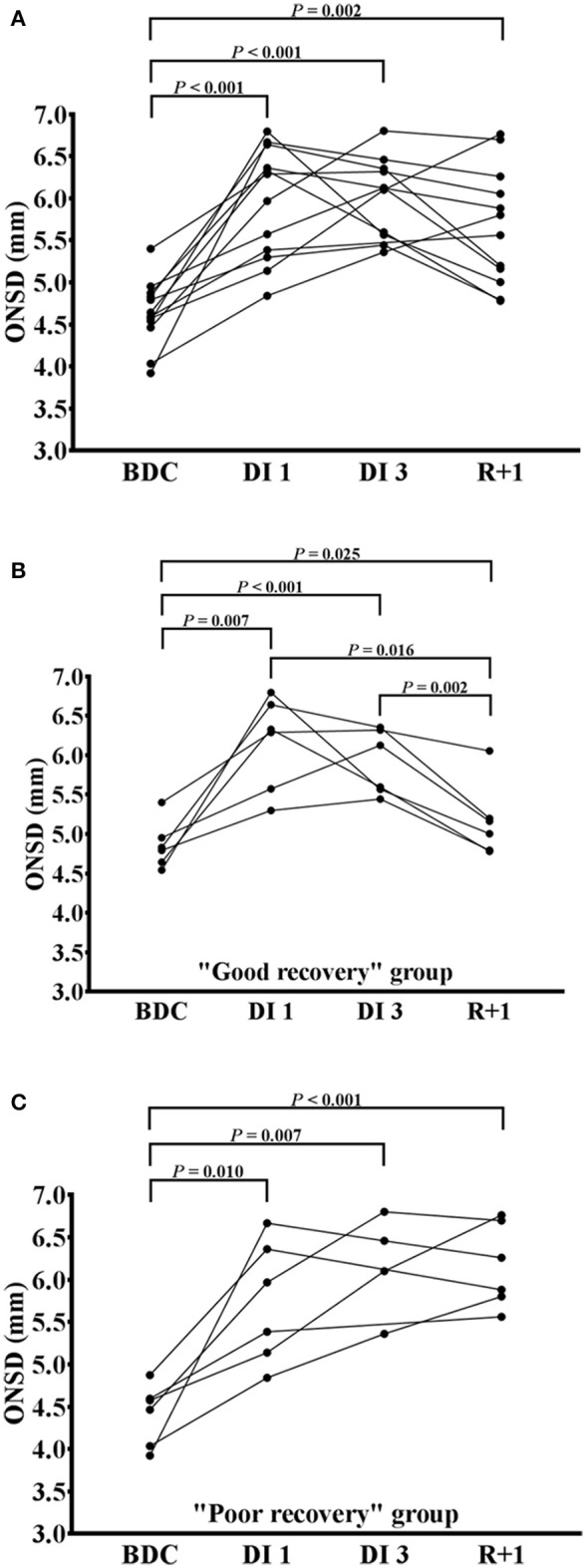
Changes in optic nerve sheath diameter after dry immersion. Changes in optic nerve sheath diameter (ONSD) before (BDC), the first day (DI 1), the third day (DI 3) and after (R+1) dry immersion in all subjects **(A)**, “good recovery” group **(B)** and “poor recovery” group **(C)**. Individuals points with the changing curves are represented. *P* < 0.05 vs. BDC; *P* < 0.01 vs. BDC; *P* < 0.001 vs. BDC.

At DI 3, measurements were only performed on 10 subjects (2 subjects were excluded for technical problems). ONSD value at BDC was the following: 4.64 ± 0.40 mm. In all subjects ONSD increased significantly by 28% during DI 1 (5.94 ± 0.67 mm; *P* < 0.001), by 30% during DI 3 (6.01 ± 0.49 mm; *P* < 0.001) and by 22% at R+1 (5.66 ± 0.69 mm; *P* = 0.002) (Figure 2A). As previously described, the 12 subjects were then allocated in “good recovery” group and in “poor recovery” group. In “good recovery” group, ONSD increased significantly by 27% during DI 1 (6.15 ± 0.59 mm; *P* = 0.007), by 21% during DI 3 (5.90 ± 0.41 mm; *P* < 0.001) and by 6% after R+1 (5.17 ± 0.47 mm; *P* = 0.025) vs. BDC (4.86 ± 0.30 mm). During R+1, ONSD was reduced compared with DI 1 (*P* = 0.016) and DI 3 (*P* = 0.002) (Figure 2B). In “poor recovery” group, ONSD increased significantly by 30% during DI 1 (5.73 ± 0.72 mm; *P* = 0.010), by 40% during DI 3 (6.18 ± 0.62; *P* = 0.007) and remained elevated by 40% during R+1 (6.16 ± 0.50 mm; *P* < 0.001) vs. BDC (4.41 ± 0.36 mm). There was no significant difference between DI 1 and R+1 and DI 3 and R+1 (respectively *P* = 0.254 and *P* = 0.410) (Figure 2C).

In VLF bandwidth, the gain and phase could not be taken into account because the coherence value was below 0.5. Phase shift was significantly elevated in LF bandwidth at R+1 compared with BDC (*P* = 0.014). None of the others transfer function analysis parameters were significantly different in VLF, LF, and HF components between BDC and R+1 (Table 3). There was no significant difference between “good recovery” and “poor recovery” groups (data not shown).

**Table 3:** Transfer function analysis of cerebral autoregulation.

	**Coherence**		**Gain (cm/s/mmHg)**		**Phase (rad)**	
	**BDC**	**R+1**	**BDC**	**R+1**	**BDC**	**R+1**
VLF	0.50 ± 0.11	0.51 ± 0.18	0.56 ± 0.22	0.48 ± 0.14	0.73 ± 0.40	1.00 ± 0.58
LF	0.64 ± 0.15	0.69 ± 0.07	0.59 ± 0.22	0.60 ± 0.10	0.82 ± 0.16	0.99 ± 0.20^*^
HF	0.63 ± 0.19	0.62 ± 0.16	0.41 ± 0.14	0.40 ± 0.14	0.52 ± 0.43	0.26 ± 0.28

Data are expressed as mean ± standard deviation. BDC, baseline data collection; HF, high frequency; LF, low frequency; R+1, 1 day after dry immersion; VLF, very low frequency;

* *P < 0.05*.

The Mxa index was significantly reduced at R+1 compared to BDC-3 (*P* = 0.009). After DI, Mxa was significantly decreased in “good recovery” group (*P* = 0.019) whereas no significant difference was noted in “poor recovery” group (*P* = 0.263) (Table 4). Mx values did not significantly different between “good recovery” and “poor recovery” groups both at BDC-3 (*P* = 0.088) and R+1 (*P* = 0.083) (data not shown). Three subjects have been excluded due to the presence of several artifacts.

**Table 4:** Dry immersion effects on overall correlation Mxa.

	**Mxa**		
	**BDC**	**R+1**	***P*-value**
All subjects	0.40 ± 0.14	0.23 ± 0.20	0.009
“Good recovery” group	0.33 ± 0.09	0.13 ± 0.10	0.019
“Poor recovery” group	0.49 ± 0.16	0.36 ± 0.24	0.263

*Data are expressed as mean ± standard deviation. BDC, baseline data collection; R+1, 1 day after dry immersion*.

**Figure 3:**
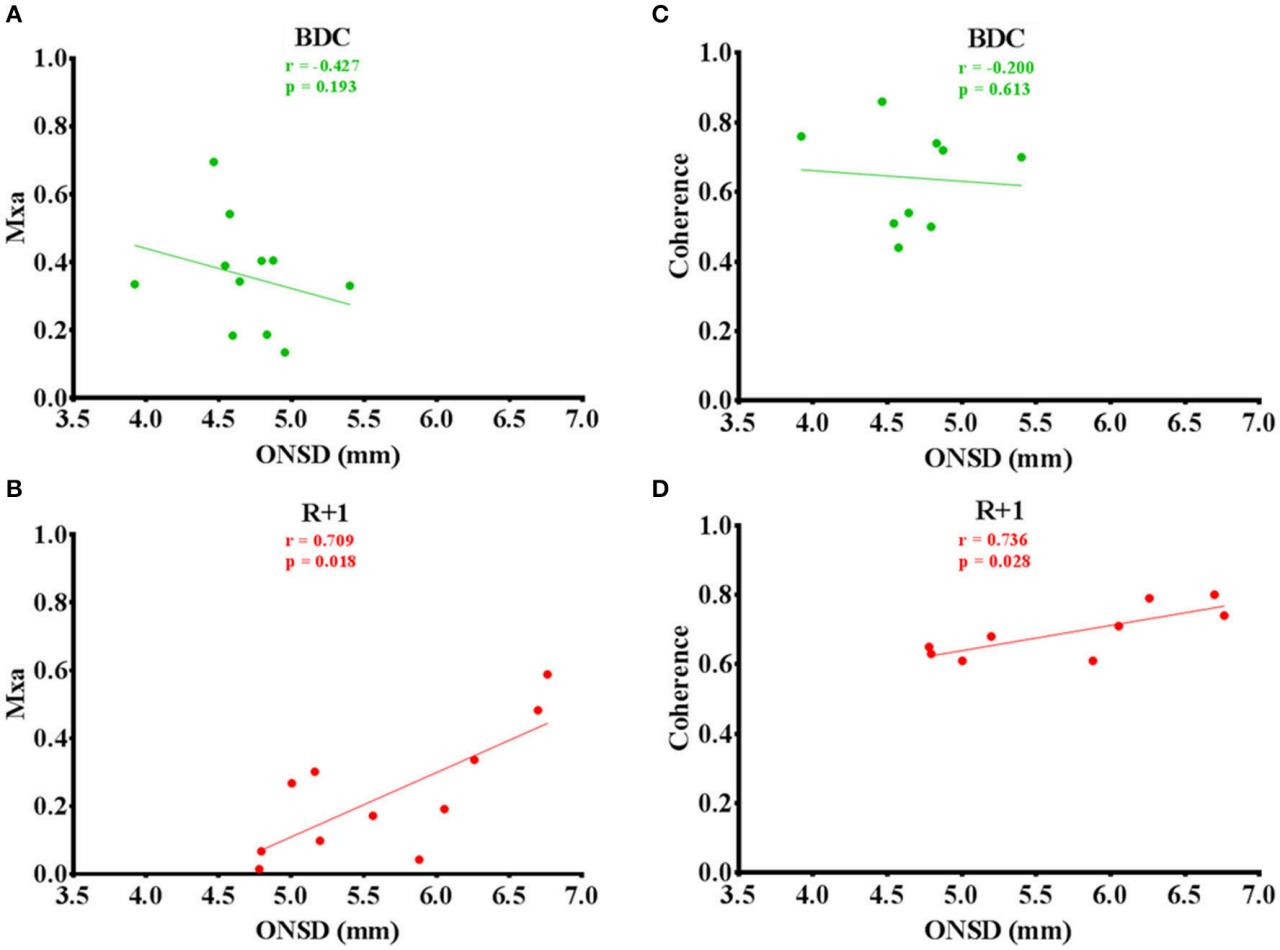
**(A–D)** Mxa and coherence values plotted versus ONSD before (green circles) and after (red circles) dry immersion with linear regression (solid lines).

At R+1, Mxa was strongly correlated with ONSD (*n* = 11; *P* = 0.018) but not at BDC (*n* = 11; *P* = 0.193). One subject has been excluded due to the presence of several artifacts (Figures 3A,B). There was no relationship between transfer function coherence in LF band and ONSD at BDC (*n* = 9; *P* = 0.613); whereas, at R+1, a high positive correlation was observed (*n* = 9; *P* = 0.028). One subject has been excluded due to the presence of several artifacts and two others subjects because the coherence value was below 0.5 (Figures 3C,D).

## Discussion

Our results indicate that DI: (1) could induce an ONSD enlargement similar to those observed in intracranial hypertension, (2) DI could improve CA, and (3) a poorer recovery of ONSD would be related to a less improvement in CA in supine position. To our knowledge, this is the first study assessing the relation between changes in ICP and CA after DI.

The reduction of the plasma volume is the result of a rapid fluid shift toward the upper body. These results are usually described in simulated microgravity (Vernikos et al., 1993). This redistribution may have other consequences and especially on ICP. Previous studies showed that the assessment of ONSD may be a good non-invasive method for detecting an elevated ICP (Geeraerts et al., 2007; Geeraerts and Dubost, 2009; Dubost et al., 2011). Our experiments reveal that DI induces an increase in ONSD with a fluctuating return to basic values. Our values are consistent to those found in other studies where 27 astronauts were exposed to microgravity (Kramer et al., 2012). A previous study demonstrated that ONSD values above 5.82 mm could reflect intracranial hypertension with a 90% probability (Geeraerts et al., 2008). Moreover, our ONSD values would be equivalent to an elevation around of 20 mmHg in ICP while the normal range is between 7 and 15 mmHg. Similar findings were found on intracranial hypertension (Geeraerts et al., 2008; Soldatos et al., 2008). One of the main factors of these changes induced by real and simulated microgravity exposure could be mainly driven by the thoraco-cephalic fluid shift. Consistently, it has been demonstrated in spaceflight that cephalad shift fluids could induce an elevated ICP (Leach et al., 1991; Heer and Paloski, 2006). However, the kinetics and the exact mechanisms involved remain unknown. This fluid shift would be the main causal factor of the ONSD enlargement and one of the underlying mechanisms would originate from direct transmission of elevated subarachnoid pressure from the intracranial to intraocular compartment through the perioptic subarachnoid space (Mader et al., 2011). Nevertheless, a recent study performed in astronauts showed that ICP measured directly, did not rise during parabolic flights (20 s microgravity period) (Lawley et al., 2017); but in contrast to our study, the ICP measurements were obtained in 90 degrees seated upright posture and obtained at the level of external acoustic meatus, which could have modified the baseline ICP values.

Most of the results of the literature are contradictory since some studies showed an unmodified, impaired or enhanced CA after actual or simulated microgravity exposure. However, the assessment of CA has never been studied during DI. In our study, we used two reference tests to assess CA the transfer function analysis and the autoregulatory index; these two methods provide information both in frequency (transfer function analysis) and time (autoregulatory index) domains. The concordant results with these two techniques strengthen our findings. The decrease in Mxa would mean that 3 days of DI could improve CA. Indeed, an increase in Mxa values reflects an alteration in CA and the threshold value of Mxa characterizing this impairment is >0.45 (Brady et al., 2010). The phase relationship between mean CBFV and mean ABP can be assessed to determine the state of CA. Nevertheless, subject's position may impact on transfer function analysis especially gain and phase functions. Indeed, significant differences could be encountered if experiments are performed in supine or seated position (Saul et al., 1989). It was previously shown that in healthy subjects, a positive phase shift was observed; whereas in patients with autoregulatory disorders, a negative phase was found (Diehl et al., 1995). In our findings, phase shift increases after DI which would mean a preserved or improved CA. These results confirm our findings for Mxa values. Moreover, a previous study reported that during short-duration spaceflight, CA could be improved in astronauts. In the LF component, gain was significantly reduced after few weeks in space compared with preflight values (Iwasaki et al., 2007). The conclusions made in our study should be done carefully. Each technique used to assess CA is different and expressed different components of the cerebral pressure-flow relationship (Tzeng et al., 2012).

One assumption is that during the simulated microgravity, relatively applicable to spaceflight, could raise the responsiveness of cerebral vascular smooth muscle to changes of transmural pressure (Iwasaki et al., 2007). It has also been shown in 14 men that the dynamic CA was improved by a reduced transfer function gain in LF range during a 2-week spaceflight (Ogawa et al., 2009). A study showed that cerebrovascular resistance slope did not significantly differ before, during and after a 7-day head-down bed rest, depicting no significant alteration of CA in 8 healthy women (Pavy-Le Traon et al., 2002). Another study demonstrated that an acute head-down tilt test in 10 healthy subjects did not modify cross-spectral analysis parameters (Cooke et al., 2003). One explanation for differences found in CA would come from, in part, time duration experience. Short term duration could enhance CA while the long term experiences may alter CA. Indeed, a previous study showed that long duration spaceflight impaired dynamic cerebrovascular autoregulation accompanied by a reduction in cerebrovascular CO_2_ reactivity (Zuj et al., 2012).

Coherence could be used to assess dynamic CA as suggested in a previous study. The intrinsic characteristic of cerebrovascular resistance implies that coherence values should be high in impaired CA situations and alternatively low in normal conditions (Panerai, 1998). Usually, CA is evaluated in VLF (0.02–0.07 Hz) and LF bandwidths (0.07–0.20 Hz) as previously described (Claassen et al., 2016). However, in our study, the VLF coherence value was below 0.5 so the gain and phase values were not sufficiently robust results. In our results, DI does not affect coherence but a positive correlation between coherence-ONSD is found, in addition to a correlation between Mx-ONSD. An altered CA is associated with head-injury with the existence of an elevated ICP (Czosnyka et al., 2001). Indeed, cerebral perfusion pressure (CPP) depends on two factors, ABP and ICP and their relationship can be established as follows: CPP = ABP – ICP. So, an elevation in ICP would imply a reduction in CPP which may provoke a vasodilation of cerebral vessels and probably a reduction in CBF (Rangel-Castillo et al., 2008). In these healthy volunteers, the enlargement of ONSD was negatively correlated with CA improvement. Little is known about the involvement of an elevated ICP on cerebrovascular remodeling and the regulation of CBF. However, a study realized in astronauts showed that cerebrovascular autoregulation could be impaired and leading to a potential syncope after their return to Earth (Blaber et al., 2011; Zuj et al., 2012).

### Study limitations

The study was performed with small numbers healthy subjects (*n* = 12) which could dampen the statistical significance of our results.

The use of transcranial Doppler necessitates the diameter of the MCA for an appropriate assessment of CBFV. This potential limitation is inherent to all CA studies using transcranial Doppler (Nasr et al., 2014).

The transfer function analysis of CA could induce bias in the findings. Phase and gain are well known to be affected by some variables (CO_2_ changes, respiration rate…). The choice of supine posture may also affect transfer function analysis; in particular gain and phase shift in the LF band compared with upright position. An increase in the sympathetic tone was described in the upright posture (Saul et al., 1989).

Despite the strong correlation existing between our dynamic CA metrics, established conclusions should be done carefully. Most dynamic CA metrics are not specifically related to each other. Each technique used would reflect different components of the cerebral pressure-flow relationship (Tzeng et al., 2012).

In conclusion, our study demonstrates for the first time that 3-day DI leads to an increase in ONSD, and enhanced the CA, with the CA improvement reversely related to the increase of ONSD. However, the kinetics underlying this process is unknown.

## Ethics statement

This study was carried out in accordance with the recommendations of “Comité de Protection des Personnes/CPP Sud-Ouest Outre-Mer I” with written informed consent from all subjects. All subjects gave written informed consent in accordance with the Declaration of Helsinki. The protocol was approved by the “Agence Française de Sécurité Sanitaire des Produits de Santé.”

## Author contributions

FL, TG, MAC, and AP carried out the experiments. MK, FL, NN, MAC, TG, MC, DA, JS, and AP evaluated results and wrote the paper.

### Conflict of interest statement

The authors declare that the research was conducted in the absence of any commercial or financial relationships that could be construed as a potential conflict of interest.
